# The relationship between illness identity and the self‐management of Inflammatory Bowel Disease

**DOI:** 10.1111/bjhp.12584

**Published:** 2022-02-03

**Authors:** Louisa Anne Peters, Emma Marie Brown

**Affiliations:** ^1^ Leeds Beckett University UK

**Keywords:** socio‐economical and psychological endpoints, illness identity, illness self‐management, inflammatory bowel disease; acceptance

## Abstract

**Objectives:**

The psychological impact of Inflammatory Bowel Disease (IBD) can be profound, leading to challenges with illness self‐management. One such impact can be an identity discrepancy, where illness identity is rejected as part of the self. The aim of this study is to examine the relationship between illness identity and self‐management of IBD.

**Design:**

A mixed‐methods approach was taken using an online survey with 167 participants living with IBD.

**Methods:**

The Illness Identity Questionnaire and Patient Activation Measure were utilized to ascertain the correlational relationship between illness identity and self‐management, triangulated with a thematic analysis of two open‐ended questions on this topic.

**Results:**

The results revealed a statistically significant relationship after controlling for possible confounders of age, illness duration, illness severity, and number of comorbidities. Positive illness identity types (acceptance and enrichment) had a moderate, positive correlation with self‐management. Negative identity types (rejection and engulfment) had a weak, negative correlation. This was supported by three main themes found from a thematic analysis and provided further insight into this relationship. Theme 1: negotiating with self as a process of acceptance; Theme 2: resigned acceptance that protects sense of self; and Theme 3: Self‐management expands from behavioural strategies to psychological processes through acceptance.

**Conclusions:**

These results suggest that the more illness is accepted into a sense of self, the better an individual is able to self‐manage IBD as more psychological resources are activated. These findings provide individuals and clinicians alike insight into utilizing identity change to improve the overall self‐management of IBD.

**Statement of contribution**

***What is already known on this subject?***

- Current self‐management strategies for Inflammatory Bowel Disease (IBD) tend to focus on behavioural strategies.
- Given the profound impact IBD can have on the sense of self, illness identity offers a novel psychological resource to improve self‐management of IBD.
- Positive illness identities have been linked to improved chronic illness management, however, research is lacking within an IBD population.

***What does this study add?***

- Demonstrates accepting IBD into identity relates to better overall illness self‐management.
- Suggests that as IBD is accepted into identity, self‐management expands from behavioural strategies to include psychological strategies.
- Adds understanding of what acceptance of illness into identity means within an IBD population.

## Background

Inflammatory Bowel Disease (IBD) encompasses the conditions Ulcerative Colitis and Crohn’s Disease, which are characterized by inflammation of the gastrointestinal tract (Yeshi et al., 2020). As a chronic condition, IBD involves life‐long self‐management, which has been linked to several health outcomes, including symptom severity, hospitalizations, and absenteeism (Saibil, Lai, Hayward, Yip, & Gilbert, 2008). From a physiological perspective, self‐management involves medication adherence to manage potential symptoms (including diarrhoea, abdominal pain, fatigue, and anaemia), as well as the adverse side effects of almost all current clinical treatment (Yeshi et al., 2020). Recent research into the lived experience of IBD has found there are also a range of negative psychological consequences including emotional turmoil, leading to low levels of self‐worth (Sammut, Scerri, & Borg Xuereb, 2015); a sense of identity loss (Matini & Ogden, 2016) such as difficulties fulfilling social identity roles (Byron, Cornally, Burton, & Savage, 2019); and the unpredictable nature of IBD symptoms have been found to have a bidirectional relationship with mental health (Mikocka‐Walus et al., 2020). Therefore, even when IBD symptoms are in remission, the condition can continue to impact the quality of life (Knowles et al., 2018). Notwithstanding this evidence, IBD self‐management tends to focus on behavioural strategies such as medication adherence, dietary changes, and managing symptoms, with the individual and their psychological needs being overlooked (Cooper, Collier, James, & Hawkey, 2010). In reality, managing chronic illness involves very little time with a clinician, and there is growing evidence that psychosocial factors play a role in managing IBD (Rogers, Kennedy, Nelson, & Robinson, 2005).

A new approach to understanding self‐management of IBD is required that goes beyond symptom management and encompasses emotional, social, and psychological well‐being. Schulman‐Green and colleagues proposed one such model of self‐management that could apply to any chronic illness and aims to illustrate the complex and interrelated processes involved (Schulman‐Green et al., 2012). Three core self‐management processes were empirically established through a meta‐synthesis spanning 49 chronic illnesses, utilizing both quantitative and qualitative data; (1) focusing on illness needs; (2) activating resources; and (3) living with a chronic illness (Schulman‐Green et al., 2012). The first process – focusing on illness needs – refers to what is traditionally considered self‐management and includes learning and behavioural strategies. The second process of activating resources refers to identifying and utilizing social, psychological, and spiritual resources relating to the individual. The final process of living with a chronic illness involves emotional processing, making meaning, and accepting the ‘new self’ (Schulman‐Green et al., 2012). Using this model, effective self‐management can be defined as a non‐linear, unique experience that involves some degree of activation of these three interrelated processes (Schulman‐Green et al., 2012). While the research into the lived experience indicates that identity can be adversely affected by IBD, there has been little research attention exploring whether adjusting identity can influence the overall self‐management of IBD.

### Illness identity and self‐management

While the Schulman–Green model of self‐management includes identity change as a contributing process to illness self‐management, it lacks explanatory detail. As such, the Identity Theory outlined by Burke and Stets was used to elucidate this relationship. Identity Theory defines personal identity as a set of meanings that characterizes the self in terms of group membership, social roles, and personal characteristics (Burke & Stets, 2009). As such, an individual possesses multiple self‐concepts that are interrelated to form an overall identity (Burke & Stets, 2009). Illness identity could be one such self‐concept developed when diagnosed with IBD. Self‐concepts provide a psychological reference to understand and perceive the context in which we live, thus influencing the thoughts, behaviours, and emotions of an individual (Burke & Stets, 2009).

The negative impact IBD can have on identity (as outlined above) can be understood as an identity discrepancy as a new illness identity is in conflict with current self‐concepts (Higgins, 1987). This can be emphasized when behaviours guided by established identities fail to counter the limitations caused by a new, forced identity (Burke & Stets, 2009). It is also possible that the intrusive nature of IBD symptoms heightens the salience of identity discrepancies (Higgins, 1987). This could result in an undesirable illness identity that can negatively impact other self‐management processes (e.g., medication adherence). It would also stand to reason that integrating an illness identity could positively influence self‐management processes. To explore this idea, Oris and colleagues established four illness identity types (Oris et al., 2016, 2018). Two positive illness identities include (1) acceptance is the degree to which illness is accepted as part of personal identity, and (2) enrichment reflects how illness has enabled personal development (Van Bulck et al., 2018). Two negative identities are defined as (1) engulfment whereby the disease dominates identity and (2) rejection of the illness as it is seen as a threat or unacceptable to the self (Van Bulck et al., 2018).

Oris and colleagues argue that the more an illness identity is integrated into a sense of self, the more an individual experience’s a sense of consistency leading to improved health outcomes (Oris et al., 2018). For example, adolescent diabetes patients who rejected their illness as part of their identity exhibited poor treatment adherence (Oris et al., 2016). Adaptive psychological functioning was also found to be impaired when diabetes patients felt their identity had become consumed by their illness (Oris et al., 2016). Furthermore, a lack of identity adjustment was found to relate to an increase in physical symptoms in patients with congenital heart disease and multisystem connective tissue disorders (Van Bulck et al., 2018). These findings indicate that illness identity influences other self‐management processes, thus impacting the overall level of illness self‐management.

### Research aim

The research reviewed above provides the following rationale for the present study. First, new approaches are needed to understand how people can better self‐manage IBD beyond behavioural strategies, and illness identity may be one means of achieving this. Second, chronic illness heightens the salience of illness identity discrepancies, thus it is crucial that people can learn to accept their illness, but more research is needed within an IBD population. Third, as identity change is indicated to be a process within illness self‐management (Schulman‐Green et al., 2012), we would expect illness identity to relate to the overall effectiveness of self‐management. Therefore, it was hypothesized that illness identity relates to illness self‐management of IBD, with acceptance and enrichment positively correlated to self‐management, and engulfment and rejection negatively correlated. Collection of qualitative data aimed to provide an individual perspective on the extent an individual feels IBD has become part of their identity, how this occurred and whether this influenced self‐management of IBD.

## Methods

### Design

A cross‐sectional, mixed‐methods study design was implemented via an online survey between April and July 2020, providing methodological triangulation to explore the phenomenon from different perspectives (Turner, Cardinal, & Burton, 2017). Self‐reported measures were used to capture the lived experiences of participants in line with the definition of self‐management as an individual experience involving a number of self‐management processes (Schulman‐Green et al., 2012).

### Participants

A total of 167 participants took part in the survey section of the study, resulting in valid data being recorded. The sample size was calculated using an estimated population of 300,000 IBD cases in the United Kingdom (Crohn’s & Colitis UK, 2020), with 80% statistical power and a 5% margin for error. The majority of participants were female (78%), with Crohn’s Disease being the dominant condition experienced (64%). The age range (19–75 years) and illness duration (1–60 years) varied widely, with the majority of participants experiencing mild symptoms or remission (72%). Full details of the participant demographics are outlined in Table 1. A total of 134 of the participants answered at least one of the qualitative questions and as such the participant demographics were very similar to those outlined above (see Table 2 for full details). Participants were recruited online via email and social media channels, with the support of IBD advocates and charities such as Crohn’s and Colitis UK and GetYourBellyOut. Ethical approval for this study was provided by Leeds Beckett University research ethics committee in April 2020.

**Table 1:** Descriptive statistics of survey participants (*N* = 167)

Age	
Mean (*SD*), years	39.8 (11.5)
Range	19–75
Gender	
Female % (*n*)	78.4 (131)
Male % (*n*)	21.0 (35)
Other % (*n*)	0.6 (1)
Disease type	
Crohn's % (*n*)	63.5 (106)
Colitis % (*n*)	34.1 (57)
Both % (*n*)	2.4 (4)
Severity	
Remission % (*n*)	36.5 (61)
Mild % (*n*)	35.9 (60)
Moderate % (*n*)	22.2 (37)
Severe % (*n*)	4.8 (8)
Duration	
Mean (*SD*), years	13.9 (11.3)
Range	1–60
Comorbidities	
Mean (*SD*)	1.9 (2.1)
Range	0–10

Note

Numbers are reported as mean (*SD*) or percentage (*n*) as indicated.

**Table 2:** Descriptive statistics of qualitative participants (*N* = 134)

Age	
Mean (*SD*), years	40 (11.6)
Range	19–75
Gender	
Female % (*n*)	76.1 (102)
Male % (*n*)	23.1 (31)
Other % (*n*)	0.7 (1)
Disease type	
Crohn's % (*n*)	66.2 (84)
Colitis % (*n*)	35 (47)
Both % (*n*)	2.2 (3)
Severity (*N* = 133)	
Remission % (*n*)	34.3 (46)
Mild % (*n*)	34.3 (46)
Moderate % (*n*)	23.9 (32)
Severe % (*n*)	5.7 (7)
Duration	
Mean (*SD*), years	13.9 (11.6)
Range	1–60
Comorbidities	
Mean (*SD*)	2 (2.1)
Range	0–10

Note

Numbers are reported as mean (*SD*) or percentage (*n*) as indicated.

### Quantitative measures

Illness identity was measured using the 25‐item Illness Identity Questionnaire (IIQ), a newly developed standardized measure (Oris et al., 2018). The scale consists of four subscales to measure each illness identity type by asking participants to indicate how much they agree with each statement on a 5‐point Likert scale ranging from 1 (*strongly disagree*) to 5 (*strongly agree*). The raw scores on each subscale were used to indicate the level of illness identity, with higher scores indicating a more dominant identity type (Oris et al., 2018). As this scale had not previously been used within an IBD population, Cronbach’s alpha analysis was carried out to establish its reliability. The Cronbach’s alpha results demonstrated that each subscale had acceptable reliability (rejection α = .748, acceptance α = .703, engulfment α = .903, and enrichment α = .916). The Cronbach’s alpha for the scale as a whole was acceptable, α = .718, further demonstrating that the IIQ measure can be reliably utilized for an IBD population.

A specific measure of self‐management developed from the Schulman–Green model could not be found. As such, the Patient Activation Measure ((PAM‐13) Hibbard, Mahoney, Stockard, & Tusler, 2005) was selected to gauge levels of self‐management, as it is an established and standardized questionnaire recommended within the National Health Service (NHS) UK (National Health Service, 2022). PAM‐13 was developed for any chronic health condition and measures the skills, knowledge, and self‐confidence an individual has in managing their long‐term illness (National Health Service, 2022). These three concepts encompass cognitive, behavioural, and psychological self‐management processes that resonate with the Schulman–Green multi‐faceted model of self‐management (Schulman‐Green et al., 2012). Participants are asked to rate each statement on a 4‐point Likert scale from 1 (*strongly disagree*) to 4 (*strongly agree*), plus 0 (*not applicable*). The raw scores were used in this study to indicate the level of illness self‐management, with higher scores indicating better self‐management of chronic illness (Luyckx et al., 2018). Control variables were also considered as factors that could have a significant effect on both illness identity and illness self‐management. These include illness duration (Mikocka‐Walus et al., 2020; Oris et al., 2018), age (Wilski & Tasiemski, 2016), illness severity (Kennedy, 2014; Luyckx et al., 2018), and comorbidities (Kerr et al., 2007).

### Qualitative methods

Two open‐ended questions were included in the survey with a free text box to allow participants to provide qualitative data on illness identity and self‐management. Question 1 asked; to what extent do you feel your IBD illness is part of your identity and how? Question 2 asked; how has this change (if any) on your identity influenced how you manage your IBD day to day?

### Analysis

Statistical analysis was performed on IBM SPSS Statistics Version 26 (2019). A Cronbach’s alpha was used to test the internal reliability of the IIQ scale, as the reliability of this measure has not been established in this population. Correlations between measure scores and the control variables were assessed for significance using Spearman’s correlation to establish relevant control variables. A two‐tailed, non‐parametric partial correlation was conducted to remove any shared variance from the control variables, producing a unique relationship between illness identity and self‐management scores (Field, 2009).

The qualitative data were then analysed using the thematic analysis method outlined by Braun and Clarke as it offers a theoretically flexible approach that can be analyst‐driven (Braun & Clarke, 2006). As a specific research question was posed for this study, codes related to the concepts of identity and illness self‐management (Braun & Clarke, 2019).

## Results

The results of the Spearman’s Rho correlation test indicated a significant relationship between all illness identity types and self‐management, as well as, each control variable significantly correlated with at least one illness identity type, self‐management, or both. Final Spearman’s Rho correlations are provided in Table 3. These results suggest that there is a significant relationship between illness identity types and self‐management (enrichment *r*_s_ = .390, *p* < .001; acceptance *r*_s_ = .344, *p* < .001; rejection *r*_s_ = −.209, *p* = .007; engulfment *r*_s_ = −.261, *p* = .001). However, the number of significant relationships with the control variables of age, comorbidities, illness severity, and duration suggests a complex relationship influenced by many factors (Table 3).

**Table 3:** Results from the Spearman’s Rho correlation analysis across all variables

Variables	Correlations (*r*_s_)								
	1	2	3	4	5	6	7	8	9
1. Age		−.032^†^	.404**	−.002^†^	−.230**	−0.1	0.054	−.223**	0.028^†^
2. Severity			−0.13^†^	0.136^†^	−0.113^†^	−0.01^†^	.187^*†^	.320**^†^	−.200*^†^
3. Duration				.163* ^,^ ^†^	0.053	.220**	−0.064	−.182*	0.152^†^
4. Comorbidities					0.022^†^	0.094^†^	−0.073^†^	.334**^†^	−0.096^†^
5. Enrichment						.485**	−.246**	−0.102	.390**, ^†^
6. Acceptance							−.437**	−0.067	.344**, ^†^
7. Rejection								−0.036	−.209**, ^†^
8. Engulfment									−.261**^,†^
9. PAM									

** Correlation is significant at the .01 level (2‐tailed).

* Correlation is significant at the .05 level (2‐tailed).

^†^ *N* differed based on whether the questions had been completed by the respondents.

To account for the overlapping variance between the control variables, a two‐tailed non‐parametric partial correlation analysis was conducted. This analysis allows for the variables of age, comorbidities, illness severity, and duration to be held constant and measure the unique relationship between illness identity and self‐management (Field, 2009). The partial correlation results showed a moderate, positive correlation, which was statistically significant, between self‐management and enrichment *r* = .371, *p* < .001, and self‐management and acceptance *r* = .331, *p* < .001. A weak, negative correlation, which was statistically significant, was found between self‐management and rejection *r* = −.171, *p* = .033, and self‐management and engulfment *r* = −.201, *p* = .012. These results suggest that as illness identity improves, so does self‐management in spite of the effects of age, comorbidities, illness severity, and duration. To verify this assumption and gain insight into how this relationship is experienced within IBD, the qualitative results need to be reviewed.

**Figure 1:**
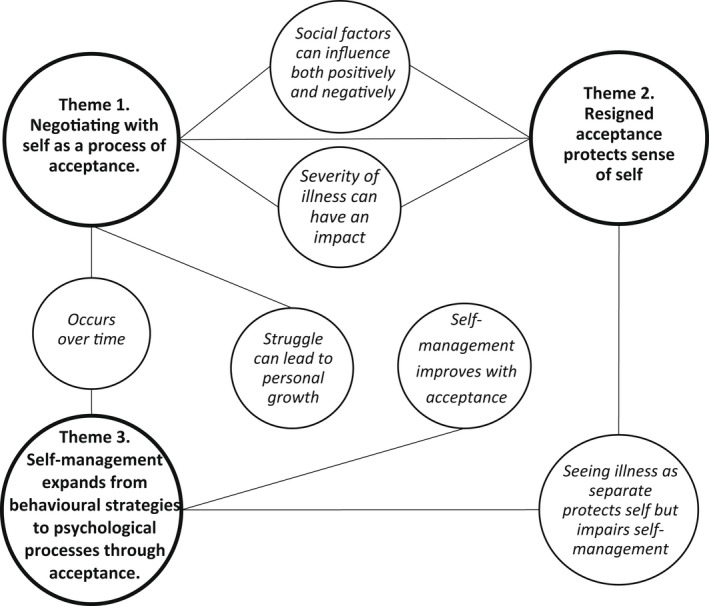
Thematic map shows the three main themes and the associated content. *Note*. The themes are labelled and highlighted in bold, while the content of these themes is displayed separately in italics with corresponding links.

A thematic analysis was conducted on the written responses to the open‐ended questions within the survey. The responses to both open‐ended questions were considered together as they interrelated. By taking an iterative approach to reading and coding the data, three main themes were developed. Figure 1 outlines a thematic map of the themes alongside their core features.

### Theme 1: Negotiating with self as a process of acceptance

When reflecting on whether IBD forms part of identity, many participants felt their identity had gone through a process of change. The key to this process was being able to negotiate with themselves and compromise to better understand how to live with IBD and accept it into their lives. This was illustrated by Alan who stated;

I have lived with IBD nearly half my life. I have sacrificed some things as a result, but I am at peace with me and my body. I push some of the restrictions it places on me at times but accept I cannot do all the things I could do without it. I can still do most things and enjoy them. (Alan, Male, Crohn’s)

The negotiation process with the self can be difficult at times with participants feeling they are fighting with their illness. However, as Catharine found, it is the emotional and psychological struggle that had led to personal growth.

…I think I've worked through a lot of the anger and resentment of having been dealt this hand of cards and am looking towards the future a little more positively. Would I wish IBD upon anyone, absolutely not ‐ have I grown as a person because of it, absolutely! (Catherine, Female, Colitis)

Social support can be an important factor in facilitating the process of negotiating with yourself, with feedback from others providing a reference to guide personal thoughts and feelings about illness. Positive social reactions and understanding seemed to facilitate acceptance of illness identity as Dana explained;

It also helps being able to talk about your illness because it helps in times of difficulty to share your thoughts and concerns with others, and it’s also enabled me to meet other people who have IBD who understand what you’re going through. (Dana, Female, Colitis)

Conversely, the more an individual accepted IBD as part of their identity, the more likely they were to have engaged with social support. For many, like Emma, this communication with others helped to create meaning from their illness by supporting others.

It glues my life together with who I am, what I do and who I know! It’s part of me and I feel strongly to be an advocate for those who are chronically ill. It has taught me so much that I am able to carry knowledge with me and help many people. (Emma, Female, Crohn’s)

### Theme 2: Resigned acceptance protects the sense of self

For many participants identity change felt forced through developing a chronic illness. This manifested as a sense of resigned acceptance of their IBD whereby they accepted having IBD but rejected it as part of their identity. Freya stated ‘When first diagnosed I was in denial that I had to adapt my life until I had a major flare. After that I conceded and have to acknowledge it exists’ (Freya, Female, Crohn’s). This separation was used as a strategy to protect their identity and was driven by fear of being consumed by their illness. This is demonstrated vividly by Grace who described their IBD not only as separate but gives it a negative persona of a rat to further distance themselves from their illness.

It is not a part of my identity full stop it is not a part of me it is a nasty horrible disgusting little rat of a thing that is separate to me in every way but happens to live inside me. I refuse to let it become a part of me. (Grace, Female, Crohn’s)

When participants felt their IBD had consumed their identity, there was a sense of loss of identity as Hannah reflects; ‘My illness has taken over my life. I can no longer work, socialise, travel or do many other things that made me, me…’ (Hannah, Female, Colitis). In addition, the negative side of social interactions, such as stigma, also had a negative influence on identity. For some, like Jo, the lack of social acceptance and labelling became a constant reminder of illness and its negative impact, which ultimately dominated personal identity.

I think having a condition which can sometimes dominate your life, while you may accept it as part of your identity it’s the people around you that don't. They start to identify you as 'the ill one' 'the always cancels plans one' and I think the first few years of being diagnosed is what I started to think [of] myself. (Jo, Female, Colitis)

### Theme 3: Self‐management expands from behavioural strategies to psychological processes through acceptance

Generally, there seemed to be an impression that self‐management improved the more illness identity was accepted. Karen noted ‘It has definitely evolved over time since being diagnosed. When I was in denial about it, I often stopped treatment. Since I accepted this disease is part of me… I’m on top of my treatments and am in remission’ (Karen Female, Crohn’s). By considering this process at each point along the illness identity spectrum we can see that not only does the level of self‐management change but also what it involves. The most distinct feature is that self‐management moves from utilizing behavioural strategies such as medication adherence to psychological strategies such as self‐compassion. At one side of the extreme, where IBD is completely rejected, there is a lack of self‐management at all. Lisa even used this lack of self‐management as a strategy to separate illness from a sense of self.

When I went to uni, I convinced myself that I was separate to my diagnosis and that I could manage my symptoms with mind over matter. I stopped taking my meds and carried on living the student lifestyle. No sleep, smoking and drinking. I went on that track for years, eventually triggering my need for emergency surgery. (Lisa, Female, Crohn’s)

For participants who expressed a sense of resigned acceptance, self‐management tended to be viewed as separate from self, purely behavioural. This reinforces the idea that IBD is a separate entity that must be dealt with, as Bill described ‘I simply managed the symptoms from a practical perspective. I do not believe it had any impact on my identity’ (Bill, Male, Colitis). However, when IBD was fully accepted into personal identity self‐management strategies utilized psychological processes such as self‐awareness and self‐compassion. Mary reflected this change was ‘Life changing as a result of listening to your body more and accepting what you can and can't do’ (Mary, Female, Colitis).

Some participants discussed improved well‐being and mental health by developing self‐compassion. This was exemplified by Colin who reflects on how IBD becoming embedded into their identity has led to a more positive outlook on self‐management.

I’m doing better now but the awful awful [time] I had has helped me love and respect my body, try to feed it better and treat it well… and be patient with myself when I can’t do things because of fatigue or other symptoms, but also be thankful of what I can do on good days coz I’ve not felt this good in years and I’ve not had this diagnosis long. (Colin, Male, Crohn’s and Colitis)

Participants’ ability to compromise with themselves to make lifestyle changes seemed to aid the process of accepting IBD into identity. This links to the first theme of negotiating with the self. Naomi discussed this interrelated process, which involved letting go of control to allow identity change to occur. This was ultimately a positive process to manage their own health.

I made a decision years ago when I changed my job position to have a stressless routine. I improved a lot. So, I better managed my disease because my identity changed: not to be resilient can save me! If it is too hard, quit! And do not feel weak but brave. [It] Is your health and life… (Naomi, Female, Crohn’s)

## Discussion

To the best of our knowledge, this is the first study to explore the relationship between illness identity and self‐management of IBD. Illness identity is understood as one possible identity that an individual can develop which to some degree is rejected or accepted within a sense of self (Burke & Stets, 2009; Oris et al., 2018). The results of this study suggest that illness identity is a concept that can be reliably measured within an IBD population. Furthermore, a significant relationship was found between illness identity and self‐management of IBD, with qualitative findings providing further insight into this relationship.

The principal findings were that enrichment and acceptance have a significant, positive relationship with self‐management, while rejection and engulfment have a significant, negative relationship. These correlations were found to be significant after controlling for control variables, indicating that illness identity influences the level of self‐management regardless of age, illness duration, symptom severity, and number of comorbidities. Such findings suggest that as illness identity becomes more accepted into personal identity, self‐management of IBD improves. While the correlational nature of this relationship does not provide any explanatory detail, the qualitative results provide some initial insight.

The qualitative data supports these findings with participants demonstrating different levels of acceptance and rejection, which seemed to influence other self‐management processes. Theme 3 captured this relationship with participants at one extreme completely rejecting their illness and not managing it at all; to the other where IBD is embedded into a sense of self resulting in a holistic view of self‐management. In addition, many participants experienced a sense of resigned acceptance (as outlined in Theme 2), whereby they acknowledged they have an illness, yet this is not accepted as part of their personal identity. For many, resigned acceptance resulted in self‐management utilizing behavioural strategies, such as medication adherence. Similar results have been found with kidney disease patients, suggesting that the defence mechanism of denial helps to protect the self (Gagania, Gemaoa, Relojod, & Pilao, 2016). This resonates with people living with IBD who expressed a fear of being consumed by their illness, and so maybe experiencing a level of denial, rather than acceptance. These findings suggest that the level of illness identity acceptance may relate to the type of self‐management processes that are also engaged.

Expanding on this idea, Theme 3 proposes that as illness identity becomes more positive, additional psychological self‐management processes are utilized. This is exemplified by participants who accepted illness into their identity by listening to their body and demonstrating self‐compassion. Theories of identity and identity discrepancy can be applied here to postulate an explanation of this process. If initial acceptance of IBD into identity relates to an increase in behavioural strategies, then identity and behaviour become congruent. Accordingly, as identity discrepancy reduces, IBD can be further integrated into identity (Burke & Stets, 2009; Higgins, 1987), resulting in an illness identity that positively influences other self‐management processes including thoughts, emotions, and psychological strategies. Such an explanation is speculative and further empirical research is needed to test these assumptions.

Another insight gained from the qualitative results relates to how illness may become accepted within identity, as suggested in the first theme of negotiating with self. Previous research has found that self‐negotiation is involved with managing IBD (Cooper et al., 2010), reflecting the interrelated nature of self‐management processes (Schulman‐Green et al., 2012). For example, participants in this study related that negotiating with themselves and making lifestyle changes resulted in illness identity acceptance. Equally the acceptance of their illness into their identity is also cited as a reason for the lifestyle change by participants. By being aware of this negotiation, individuals, and those who support them (such as clinicians), can actively engage in this process to encourage acceptance of illness identity, and improve overall illness self‐management. It is clear from these initial findings that future research needs to explore the relationship between illness identity and other self‐management processes to establish any connections and potential predictive relationships.

Lastly, both the quantitative and qualitative results revealed that variables such as age, illness duration, symptom severity, and number of comorbidities are related to either illness identity, the overall level of self‐management, or both. However, the influence of control variables was inconsistent within both the correlational analysis and participant feedback. Yet this finding in itself could indicate that the activation of self‐management processes is dependent on the individuals’ context. This concept is supported by the social factors reported in this study. For some participants negative or stigmatized social reactions were internalized, developing a negative identity (Haslam, Jetten, Cruwys, Dingle, & Haslam, 2018). In contrast, those who talked about their IBD experience created positive meaning through helping others. This can be understood by concepts within social identity theory, whereby social interactions can be used as a resource for defining one’s own identity through comparing self to others and developing self‐concepts in relation to others (Burke & Stets, 2009; Haslam et al., 2018). Stigma has been found to link closely with negative health outcomes through increased physiological stress responses and the higher probability of engaging in unhealthy‐coping behaviours (Haslam et al., 2018). Therefore, accessing positive social resources to counter the effects of stigma presents a mechanism to improve illness identity (Schulman‐Green et al., 2012). The findings from the current study suggest different self‐management processes such as social resources, self‐negotiation, and illness identity, can interact to affect the overall level of self‐management of IBD.

### Limitations and future research

First, the majority of participants in this study were female, and most had experienced Crohn’s disease, which may have affected the results. Including these factors within future analysis would enable any effect to be controlled for. Second, both measures used within this study relied upon self‐reported responses and does not offer a comparison to objectively defined self‐management processes, such as medication adherence. Self‐reported measures were selected as the most appropriate tool for this study to capture the lived experiences of participants, and the cross‐sectional study design. Objective data sources could be included in future studies to provide another perspective of self‐management processes.

Lastly, the findings of this study highlight the complex processes involved in the self‐management of IBD and suggest that identity change may be an influential self‐management process. However, further research is needed to establish if and how illness identity relates to other self‐management processes. For example, the processes outlined in the Schulman–Green model, and in particular those highlighted by the qualitative findings of this study, such as self‐negotiation, and access to social resources. Specific therapy techniques, such as cognitive analytic therapy, could be explored as a support option for IBD patients to aid the acceptance of illness identity, having shown promise within other chronic conditions (Tilden, Charman, Sharples, & Fosbury, 2005).

### Conclusion

In conclusion, the present study provides initial evidence that illness identity appears to be an influential self‐management process for IBD. The findings suggest that accepting IBD into identity could improve the overall self‐management of IBD. By acknowledging the process of identity change, individuals and health care practitioners can be equipped with another psychological resource to improve IBD self‐management. A better understanding of how illness identity relates to other self‐management processes such as self‐negotiation and social resources could aid the improvement of self‐management programmes or interventions.

## Conflicts of interest

The authors declare no conflict of interest.

## Author contribution

**Louisa Peters:** Conceptualization (equal); Data curation (equal); Formal analysis (equal); Investigation (equal); Methodology (equal); Project administration (equal); Resources (equal); Software (equal); Validation (equal); Visualization (equal); Writing – original draft (equal); Writing – review & editing (equal). **Emma Marie Brown:** Conceptualization (equal); Formal analysis (equal); Methodology (equal); Resources (equal); Software (equal); Supervision (equal); Visualization (equal); Writing – original draft (equal); Writing – review & editing (equal).

## Data Availability

The authors confirm they have full control of all primary data and that they agree to allow the journal to review their data if requested. Primary data can be made available upon publication.
